# Design and Deployment of Low-Cost Sensors for Monitoring the Water Quality and Fish Behavior in Aquaculture Tanks during the Feeding Process

**DOI:** 10.3390/s18030750

**Published:** 2018-03-01

**Authors:** Lorena Parra, Sandra Sendra, Laura García, Jaime Lloret

**Affiliations:** 1 Instituto de Investigación para la Gestión Integrada de Zonas Costeras, Universitat Politècnica de València, 46022 Valencia, Spain; loparbo@doctor.upv.es (L.P.); laugarg2@teleco.upv.es (L.G.); 2 Departamento de Teoría de la Señal, Telemática y Comunicaciones, ETS Ingenierías Informática y de Telecomunicación, Universidad de Granada, C/Periodista Daniel Saucedo Aranda, s/n, E-18071 Granada, Spain; ssendra@ugr.es

**Keywords:** aquaculture, feeding process, water quality, physical sensors, wireless sensor network

## Abstract

The monitoring of farming processes can optimize the use of resources and improve its sustainability and profitability. In fish farms, the water quality, tank environment, and fish behavior must be monitored. Wireless sensor networks (WSNs) are a promising option to perform this monitoring. Nevertheless, its high cost is slowing the expansion of its use. In this paper, we propose a set of sensors for monitoring the water quality and fish behavior in aquaculture tanks during the feeding process. The WSN is based on physical sensors, composed of simple electronic components. The system proposed can monitor water quality parameters, tank status, the feed falling and fish swimming depth and velocity. In addition, the system includes a smart algorithm to reduce the energy waste when sending the information from the node to the database. The system is composed of three nodes in each tank that send the information though the local area network to a database on the Internet and a smart algorithm that detects abnormal values and sends alarms when they happen. All the sensors are designed, calibrated, and deployed to ensure its suitability. The greatest efforts have been accomplished with the fish presence sensor. The total cost of the sensors and nodes for the proposed system is less than 90 €.

## 1. Introduction

Aquaculture is a promising option to offer sustainable fish meat, and it will allow a considerable increase of fish consumption by 2050 [1]. It can be implemented under different conditions. Some facilities are deployed in the sea, while other facilities are placed inland. In inland facilities, fish are kept in tanks that can vary in size and materials. In facilities that perform intensive aquaculture, many efforts are taken to maximize the performance of the fish. Fish performance depends on different factors: environmental factors [2], production factors [3], and biotic factors [4]. In the facilities, it is possible to control some of those factors. Halogen lights are used over each tank to provide illumination to the tanks. With light, it is possible to change the photoperiod and modify the behavior of the fish to enhance their performance [5]. Moreover, filters can be used at the water entrance to eliminate the turbidity. Thus, the negative effects of turbidity are reduced, improving fish performance [6]. Other factors such as water temperature or water conductivity are not usually modified, although it is possible to modify them. Water temperature and conductivity can change the feeding needs of fish kept in the tanks [2]. Moreover, if fish are stressed their feeding consumption falls and the performance decreases. Many factors can cause stress on fish. There are many parameters that must be monitored to help fish enhance their performance and therefore improve the sustainability and profitability of the fish farms.

In recent years, the development of different Information and Communication Technologies (ICT) in conjunction with the creation of low-cost small sensors have made it possible to monitor many processes. Wireless sensor networks (WSN) are a clear example as they are often used for farming purposes. WSN have been used for monitoring the three vigor [7], greenhouses [8] and citrus crops [9]. Moreover, WSN are employed to monitor the state of farm animals such as goats [10] or cows [11]. Some systems have been proposed for monitoring fish farms [12,13,14,15,16,17,18,19,20,21,22]. They will be analyzed individually in the related work section. The majority of them are based on monitoring water quality including just a couple of water parameters to be monitored. Moreover, they usually employ commercial probes. The commercial probes for underwater monitoring have a high cost. Thus, if a WSN were to be utilized to monitor several parameters using commercial probes in all the production tanks, the cost of the system would be unaffordable for the fish farms. Moreover, other authors propose systems for monitoring fish behavior [23,24,25,26,27,28,29,30]. In the related work section, we will analyze each proposal. Therefore, if we aspire to measure different parameters in fish farms facilities with WSN, it is crucial to reduce the cost of the sensors and include a wider variety of parameters in the same system.

The aim of this paper is to design and deploy a low-cost WSN to monitor fish feeding process and water quality in aquaculture tanks. The system is composed of sensors that measure different parameters of the water quality (such as temperature, turbidity, or conductivity, among others), of the tank conditions (such as illumination and water level), and of the fish feeding behavior (such as swimming depth and velocity and fallen pellets). Moreover, the system has other sensors, such as the humidity sensor, that actuates as an emergency turn-off system to prevent damages caused by water in the node and other electronic circuits. In addition, presence sensors are placed in each tank to control the possible effects in fish behavior of the passage of the workers near the tanks. A total of three nodes control the different parameters in each tank. The nodes are wirelessly connected to an Access Point (AP) that sends the data to a server. The data is available on the local area network and on the Internet. The system can send alarm messages to different workers if abnormal situations are detected.

The rest of paper is structured as follows. Section 2 presents the state of art of the WSN for fish farm monitoring and fish behavior monitoring. The proposed system is shown in Section 3. Section 4 details the results of the calibration and operation of our system. Finally, the conclusions and future work are presented in Section 5.

## 2. Related Work

In this section, the state of art of the systems for fish farm monitoring is shown. Several authors propose different systems for water quality monitoring.

Water quality monitoring and fish behavior monitoring are crucial to improve the efficiency of aquiculture. In this section, the related work on the available water quality monitoring systems and some of the research performed on fish behavior monitoring is presented.

Wireless Sensor Networks (WSN) have become a solution for performing water quality monitoring. Francisco J. Espinosa-Faller et al. presented in [12] a WSN-based water monitoring system. The system employed ZigBee to transmit the information gathered by the sensors from the recirculating system. Then, the information was stored in a MySql database. Temperature, pressure, and dissolved oxygen was measured throughout the day. When a problem was detected, an SMS or an E-mail was forwarded to alert the person responsible for the facility. Another WSN-based water monitoring system was presented by Mingfei Zhang et al. in [13]. The system was able to measure pH, water temperature, water level and dissolved oxygen. The data was forwarded to a database that provided the information to the software to be monitored in real-time. The software was developed employing Visual studio 2005 and separates logic, display and data layers to improve scalability and reusability. Lastly, warnings were forwarded via SMS or graphical device twinkle to the users. WSN is employed as well for the recirculating monitoring system proposed by Qi Lin et al. in [14]. The system was composed of a three-layer architecture comprised of the remote layer, the server layer and the client layer that obtained, transported, and displayed the information gathered on water temperature, conductivity, salinity, and pH. Moreover, a software solution was developed as well to monitor the obtained information by accessing a Postgre SQL server employing a WLAN (Wireless Local Area Network). Huang Jianqing et al. presented in [15], a WSN-based water quality monitoring system that gathered data on pH, water temperature and dissolved oxygen. A real-time interface allowed the display of the data numerically and graphically. Several testing results were provided displaying fluctuations during the day. These results show a 1.40% of relative error for pH, 0.27% for temperature and 1.69% for dissolved oxygen. The water quality monitoring system presented by Daudi S. Simbeye et al. in [16] measured pH, water level, water temperature, and dissolved oxygen and employed ZigBee to forward the data. They used the LabView software (National Instruments Corporation, Austin, TX, USA) to display the obtained information. Furthermore, several experiments regarding communication performance, battery performance and sensor readings were performed over a period of six months. Results showed the fluctuations of these parameters throughout the day. Xiuna Zhu et al. designed in [17] a water quality monitoring system for fish farms. It employed artificial neural networks (ANN) to forecast water quality to prevent losses. The data acquisition node measured water and room temperature, percentage of dissolved oxygen saturation, dissolved oxygen concentration, pH, electrical conductivity, and salinity employing a variety of sensors. The data was then forwarded to a server to be remotely accessed. Cesar Encinas et al. proposed in [18] an IoT-based water monitoring system for aquaculture. They employed an Atlas Ph Probe digital sensor (Atlas Scientific, Long Island City, NY, USA), an analog temperature sensor and an Atlas Dissolved Oxygen Probe as well as an Arduino node that employed a ZigBee module to forward the information to a MySQL database. Moreover, the system utilized 200 mA/h rechargeable batteries and was able to perform for 8 h. The information was able to be visualized through a desktop or a mobile application. Soonhee Han et al. designed in [19] an environment monitoring system specific to aquaculture farms. They measured water quality, fish food and the medication provided to the fish. For water monitoring, they employed sensors to achieve data on conductivity, temperature, dissolved oxygen, and air saturation. The user interface allowed monitoring of the parameters from a graph and a log. Furthermore, the system was able to alarm users when the fish disease was propagated to provide treatment. Gianni Cario et al. presented in [20] a water quality monitoring system for fish farms. The system was composed of SUNSET (Software Defined Communication Stack) for networking purposes and Hydrolab Series 5 probes for data acquisition. The measured parameters were temperature, pH, luminescent Dissolved Oxygen (LDO), salinity, Oxidation Reduction Potential (ORP) and specific conductance (SpCond). Moreover, energy consumption was reduced employing new sleep and wake-up mechanisms. Another water monitoring system was presented by Luo Hongpin et al. in [21]. Their system employed ZigBee and GPRS modules with a STM32F103 chip. They also employed a Pt1000 temperature sensor and a YCS-2000 dissolved oxygen sensor as well as other sensors to measure pH and ammonia. The obtained information could be monitored with a computer program developed with Labview. This program allowed monitoring of each parameter in a separate graph as well as numerically. Results showed a packet loss rate of 0.43%. Yang Shifeng et al. proposed in [22] an aquiculture environment monitoring system that employed RF (Radio Frequency) and GSM (Global System for Mobile communications) to measure temperature and dissolved oxygen. Two algorithms were designed to determine the performance of the monitoring center and the different substations. Substations performed a 24-h uninterrupted data acquisition and forwarded the information in real-time.

The techniques employed to monitor fish behavior usually utilize optic and acoustic techniques. Vassilis M. Papadakis et al. implemented in [23] a fish behavior monitoring system that was able to monitor 9 fish tanks at the same time employing computer-vision. They evaluated stock density as a stress factor. Their system was able to be controlled remotely and provided real-time images of the fish. The obtained results showed a significant statistical difference in treatment comparison only for the experiment performed with the undamaged mesh. A survey of other existing vision-based systems for fish behavior monitoring was presented by Mohammadmehdi Saberioon et al. in [24]. They defined the two major areas of applications for optical sensors as pre-harvesting and during cultivation, and post-harvesting situations. They also performed a discussion on fish monitoring technologies such as machine vision, hyperspectral imaging, thermal imaging, and x-rays. They summarized the applications of optical sensors for fish monitoring into five types being fish sorting, fish quality, physical attributes, chemical attributes, and food security. A monitoring behavior system based on a flat passive integrated transponder antenna array was presented by J. D. Armstrong et al. in [25]. They employed their system to record the movements of salmon shoals. Several experiments were performed employing different quantities of salmons for each test. The obtained results showed a success rate higher than 99%. Moreover, fish presented no unusual behavior to the antenna array. Stéphane G. Conti et al. employed acoustics for monitoring fish behavior, growth, and density, as described in [26]. Their experiments were performed over sardines, rockfish and sea bass deployed in tanks. They employed the scattering cross section to determine the behavior of the fish and to trig an alarm when anomalies were detected. They were able to monitor the growth-rate of the fish as well employing a first-order polynomial equation and a second-order polynomial equation. An imaging sonar referred as DIDSON (Dual-frequency Identification Sonar) was utilized by Hui Zhang et al. in [27] to monitor swimming pattern and the length of Chinese sturgeons. Experiments were performed with over 2500 targets. Authors were able to find a relation between swimming pattern and body length from the obtained data. The lengths detected by DIDSON were 35.6% shorter than the ones obtained from manual measurements. Moreover, results showed that fish mostly swam in a circular motion and close to the net. B. P. Ruff performed stereo Image analysis to monitor fish in [28]. They were able to measure shape, size position and spatial orientation of a fish. Three different experiments were performed in fish tanks with a depth of 3 m and 1 m of cross-section. All experiments had four stages being calibration, image acquisition, identification of the measurement points and 3D position calculation. Results showed an error between 3% and 5% for determining the length of the fish. A GPS tracking device was employed in [29] by David W. Sims to monitor sunfish Mola mola. The system was able to monitor the position with an approximate accuracy of 70 m. Experiments were performed on three different fish with sizes varying from 0.6 m to 1 m. The route performed by the fish was able to be visualized in a map. Moreover, Data on the speed of the fish was acquired and displayed on graph locating each measure on the map by adding a dot on the route. D. Karimanzira et al. presented in [30] a guidance system for a fish behavior and water quality monitoring system that employed an autonomous underwater vehicle. The vehicle was provided with a conductivity and oxygen optode sensor, as well as a spectrometer. LED cameras were also attached to the vehicle to observe fish behavior. The vehicle was able to navigate through the cages without colliding with them. As a result, the vehicle was able to obtain information without any collision.

The systems for water quality monitoring measure the same parameters not considering other important factors to monitor such as the turbidity of the water. Moreover, many of the papers do not specify which sensors they have utilized, or they employ expensive sensors, resulting in a high-cost system that is difficult to implement in fish farms with few resources. Furthermore, fish behavior monitoring is not usually incorporated in the same system increasing the investment owners must do to improve the efficiency of their fish farm. In this paper, we present a low-cost water quality and fish behavior monitoring system. The system can measure the water quality parameters, tank state factors and fish behavior during the feeding process.

## 3. Materials and Methods

In this section, the developed system is detailed. First, the architecture of the proposed system is presented. Second, the employed physical sensors and its conditioning circuits are described. Then, the node and its configuration are presented.

### 3.1. Architecture

In this subsection, the architecture of the proposed system is shown. The system is based on sensors for monitoring 10 parameters, including water quality parameters, tank parameters, fish behavior and humidity inside the node boxes. In each production tank, there are three nodes monitoring different parts of the tank. The employed nodes send the data wirelessly to the AP.

**Figure 1:**
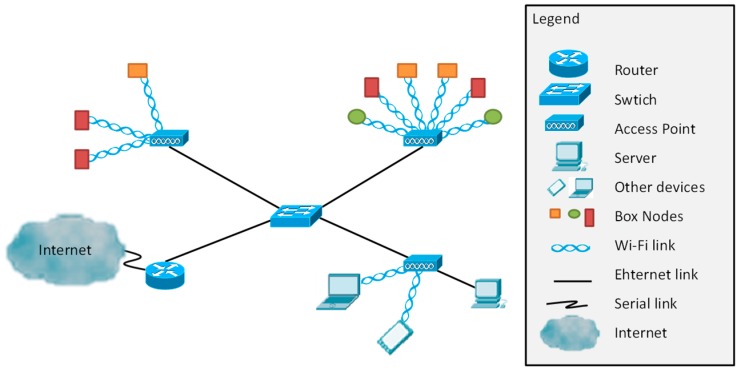
Network topology proposed.

First, the physical topology is shown, see Figure 1. In the fish farm, a local area network will be deployed. Different box nodes (BN) are placed in several points of the facilities. The nodes are wirelessly connected to the AP using Wi-Fi technology. The APs are connected to the switch with an Ethernet link. Several APs are located at different points of the facilities; some of them are placed in the rooms with the production tanks. However, other APs are placed in the offices and those AP do not send data to the database. Those AP are used to send data to the workers if any alarm messages are generated. Finally, the switch is connected to a router to have internet access with a serial connection. The topology is based on an extended star topology.

**Figure 2:**
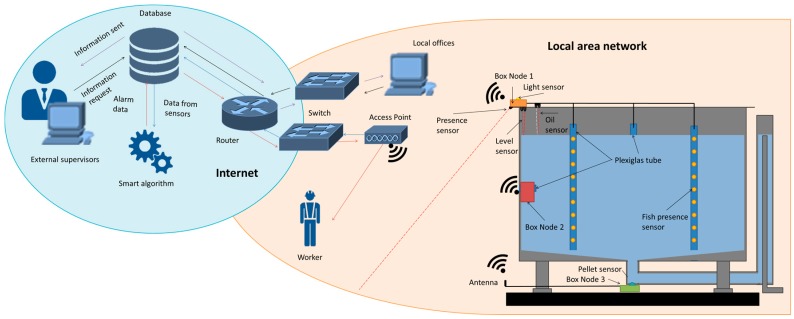
Architecture of the proposed system.

Figure 2 shows the architecture of the entire system, including the database and the smart algorithms applied in the cloud. To save the data and move the computing activity to the cloud, it is possible to opt for a commercial solution such as Amazon Web Services [31] or Microsoft Azure [32] which implies an additional cost. In our case, we have used an own external web server which can be accessed through the internet. Besides, supervisors who are not located in the fish farm facilities can make an information request to the database from anyplace and anytime. Furthermore, in Figure 2 it is possible to see the location of the sensor and BN in the aquaculture tank. The BN 1 contains the following sensors: illumination, water level, oil layer, workers presence and fish behavior sensors. The BN 2 contains the rest of the water quality sensors, which include a temperature senor, turbidity, and conductivity sensor. Finally, the BN 3 contains the feed fallen detector. In addition, all the BN contains and humidity sensors. In normal condition, the nodes sense and send the data to the database. Once the data arrives at the database, the smart algorithm is applied. If the smart algorithm concludes that the data corresponds to normal situation no further actions are done. Nevertheless, if the smart algorithm concludes that the data does not fit with the normal situation, an alarm message is sent to the designed worker with the necessary information.

**Figure 3:**
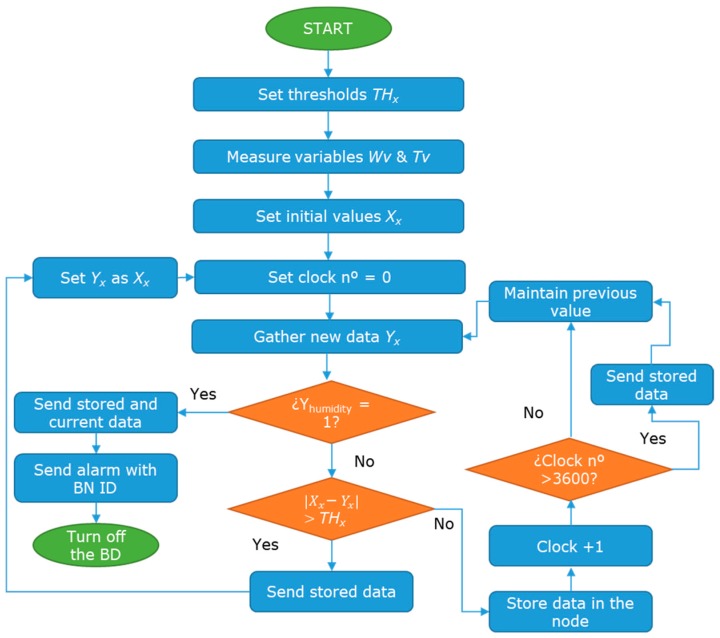
Operation algorithm for the water variables and tank variables.

To avoid the energy waste in the sending data, algorithms can be used to send only the relevant data. Thus, we reduce the number of information send reducing therefore the energy consumption in the data transmission. The energy consumption is one of the main deals in the WSN [33] and many protocols have been developed to reduce the energy waste [34,35]. First, the thresholds for each variable are set (*TH_x_*). Next, the sensor gathers data and save this data as reference value *X_x_*. A reference value is set for each one of the water variables (*Wv*), including temperature, conductivity, turbidity, and oil layer, and for the Tank variables (*Tv*), which include water level, illumination, and workers presence. The next step is to set a clock time = 0. Following, the systems gather new data and it is named as *Y_x_.* Then, the system checks the data from the humidity sensor. If the data is equal to 1, which indicates that there is humidity in the BN, then the system sends all the stored data; send an alarm to the workers and turn off this BN. If the humidity sensor gives a value of 0 the operation continues. The *Y_x_* ta is compared with the *X_x_*. If the difference is higher than the *TH_x_*, the stored data is sent to the database. Moreover, the *Y_x_* is set as *X_x_*, the clock is set again to 0 and the operation algorithm gathers new data. If the difference between them is not higher than the *TH_x_* the *Y_x_* is stored in the SD card of the node and the clock adds one counter more. If the clock value is higher than 3600 (1 h) all the data is sent to the database. If the clock value is lower than 3600 the *X_x_* data are maintained, and the algorithm follows gathering new data. The entire algorithm can be seen in Figure 3.

**Figure 4:**
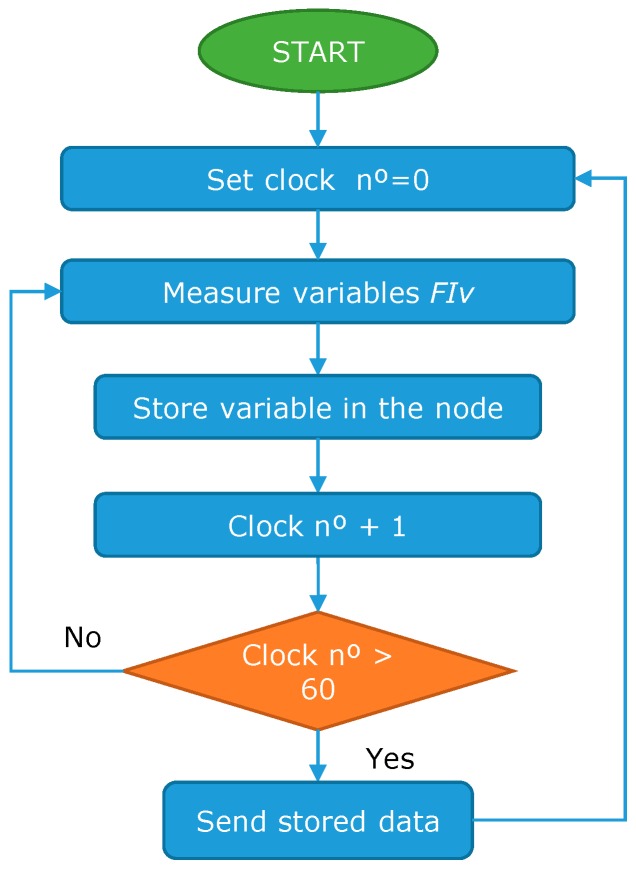
Operation algorithm for fish behavior algorithm.

**Figure 5:**
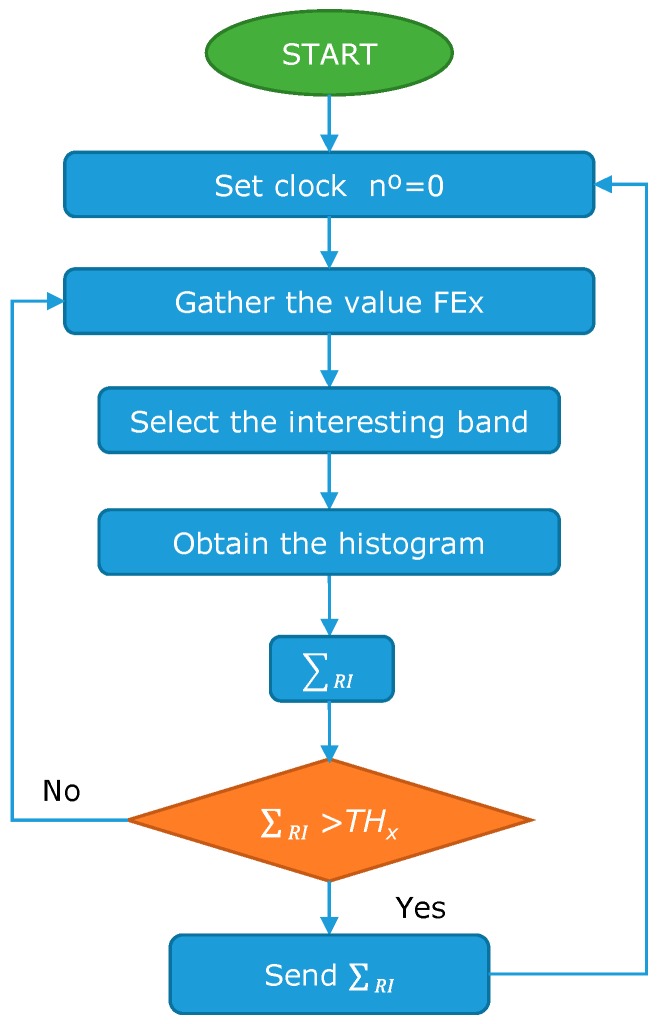
Operation algorithm for feed falling sensor.

Another algorithm is used for the fish behavior (*FI_x_*) sensors, see Figure 4. In this case, the node stores the data for 1 min and then sends all the data to the database. Finally, for the data from the feed falling (*FE_x_*) sensor, the algorithm shown in Figure 5 is applied. Once the data from the sensor is gathered, the data about the interesting band is selected and the data from the other two bands are discarded. Then, the histogram of the selected band is obtained. Finally, the summation of the region of interest (RI) is done. If the summation of the RI is higher than the established *TH_x_* the data is sent. If not, the algorithm continues gathering new data.

### 3.2. Sensors

In this subsection, the sensors included in our system are presented. Different types of sensors, including optical sensors, thermal sensors, and magnetic sensors, among others, are used. The sensors are described in three sections. First, the sensors for monitoring water quality are presented. Next, the sensors for cage environment monitoring are shown. Lastly, we describe the sensors for fish feeding behavior.

#### 3.2.1. Water Parameters

For water quality monitoring in fish farms, the physic-chemical parameters monitored by our system are temperature, conductivity, turbidity, and the presence of an oil layer in the surface of the water. As it is explained above, to create a low-cost system, the price of the sensors is a limiting factor. For this reason, we create our own sensors based on electronic components with low-cost.

**Figure 6:**
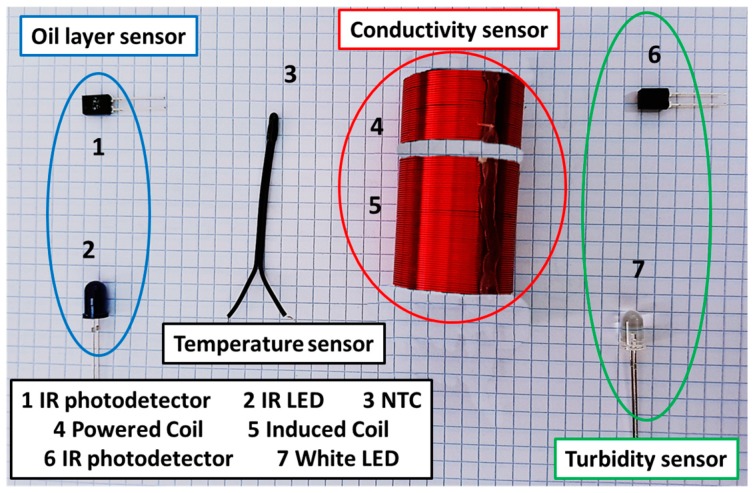
Employed components for sensor to monitor the water quality.

First, we describe the employed sensor for temperature monitoring. There are several options for temperature sensing. The most employed ones for water monitoring are the RTD and the thermistors. The reasons why these are the most used sensors are their high working range, adequate accuracy, and low price. In our case, a thermistor type negative temperature coefficient (NTC) was employed. The NTC presents greater resistance when temperature decreases. The other advantage of the NTC is the linear relationship between resistance and temperature. The employed NTC is the NTCLE413E2103F520L from Vishay [36]. The price of this sensor is 0.96 €. The working range of the sensors according to the datasheet is −40 °C to 105 °C. Our working range will be from 5 °C to 35 °C. The employed NTC can be seen in Figure 6.

Following, the conductivity sensor is presented. As for temperature sensing, different options can be used. In this case, and considering that one of the main requirements is to avoid the contact between the sensing element ant the water, we propose the use of inductive sensor. This sensor is composed of two copper coils. One of the coils is powered by a sine wave. The generated electromagnetic field change depending on the conductivity of the water. The secondary coil, depending on the magnetic field, induces an electric voltage. The employed sensor was described by Parra et al. [37,38]. The coils were done with copper wire with 0.4 mm of diameter. The coil diameter was 25 mm. The powered coil has 40 spires and the induced coil 80 spires. The induced voltage increases when the conductivity of the water increases. In a previous test performed with these coils a PVC tube was used [37,38]. Nevertheless, in this case, a crystal cylinder will be used. The working range of the sensor goes from 0 mS/cm to up to 80 mS/cm. In the marine fish farms, the expected salinity goes for 0 mS/cm to 29 and to 38 mg/L [39], which corresponds to a range from 44.9 to 57 mS/cm. The conductivity sensor included in our system can be seen in Figure 6.

The turbidity sensor is described below. There are two main methods to measure the turbidity, the acoustic and the optical method. In this case, the optical method is selected. The employed sensor is described in [40]. It is composed of an infrared (IR) light emitting diode (LED) and an IR photodetector. The employed IR LED is the TSHG6200 from Vishay [40], the peak wavelength of this LED is 850 nm. Moreover, the employed IR photodetector is the BPW83 from Vishay [41]. Its range of spectral bandwidth goes from 790 to 1050 nm and its wavelength of peak sensitivity is 950 nm. The IR photodetector offers a fast response, the resistance of the photodetector decreases when the turbidity increases. The relationship between turbidity and resistance is linear in the range from 0 to 250 mg/L. If we intend to include higher values, the relationship changes to other type of relation. However, the expected values of turbidity in fish farms are low. Only in adverse conditions the turbidity values can increase. The turbidity sensor can be seen in Figure 6.

The last sensor that monitors the water quality is the oil sensor. This sensor must be able to detect the appearance of an oil layer in the surface of the water. This sensor is based on the model proposed by Parra et al. [42]. A white LED is employed to illuminate the water surface and IR photodiode is used to measure the light emission. The employed white LED is the VLHW4100 from Vishay [43], its wavelength emission goes from 400 to 700 nm and the peak wavelength is placed at 450 nm. The photodiode is the BPW41N from Vishay [44]. The range of spectral bandwidth goes from 870 to 1050 nm with a peak at 950 nm. The photodiode is not the same that was used in [42] but their characteristics are the same. Unlike the other sensor, the oil layer sensor is not a quantitative sensor. This sensor is only able to determine the presence of an oil layer, but it is not possible to quantify the thickness of the oil layer. The oil sensor can be seen in Figure 6.

#### 3.2.2. Tank Parameters

Some parameters of the tank and its environment, such as water level, illumination, and the presence of workers near the tank, must be monitored. As in water quality sensors, the sensors employed for tank control are simple electronic components sensitive to different light wavelength combined in different ways.

**Figure 7:**
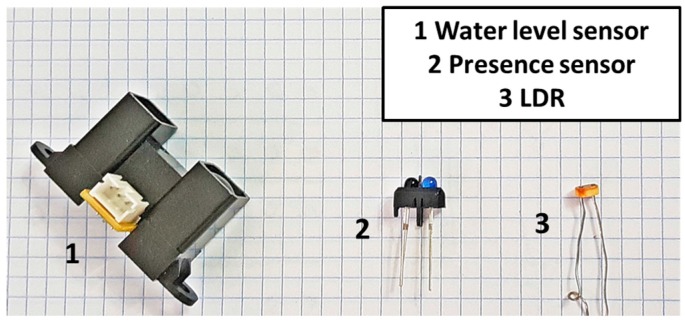
Employed components for sensor to monitor the tank.

First, the water level sensor is described. There are two main options to measure the level of the water, employing acoustic sensors or optical sensors. To measure the water level, a level sensor based on IR emission and reception was selected [45]. This sensor was already tested for fish farms in [38]. The measurement ranges from 20 to 150 cm. This sensor is already calibrated; the maximum output voltage corresponds to the minimum distance, 20 cm. However, the maximum voltage is too high to be connected directly to the node. For this reason, it is necessary to add a conditioning circuit to reduce the maximum voltage. The sensor can be seen in Figure 7.

Following, the sensor for monitoring the illumination in the tanks is presented. To measure the luminosity of the halogens that illuminates the tank there are different options. All the options are based on optical sensors. We select to use a light dependent resistance (LDR) for monitoring the illumination, see Figure 7. We select the LDR because it has a low price and it provides a fast response. The resistance of the LDR increases with the light intensity. The selected LDR to measure the illumination in the tanks is the NORPS-12 from Advanced Photonix [46]. It is encapsulated in a humidity resistant coating. Moreover, it is enclosed in a plastic casing. The minimum and maximum resistances are 5.4 kΩ and 1 MΩ. Finally, the sensor for detecting the presence of workers near the tanks is shown. This sensor is based on the IR light emission and reception. The system is similar to the sensor for water level. It has an IR emitter and IR receiver. The sensor can be seen in Figure 7.

#### 3.2.3. Feeding Parameters

For monitoring the feeding parameters, the fish behavior and the falling pellets must be measured. Again, low-cost components must be used to ensure the low-cost of the system.

Firstly, the system for monitoring the fish behavior is described. The system is composed of three transparent Plexiglas tubes placed in different points of the inside of the tank. Inside each tube, there are 9 LDR sensors placed at 15 cm from each other. The operation of this sensor is based on the light refraction caused by the fish scales when they are swimming. Most of the currently available systems are based on the use of acoustic waves or machine vision. However, the systems based on acoustic waves have high energy consumption. On the other hand, the systems based on machine vision require from high computation. Furthermore, the systems based on machine vision usually do not give information about the swimming depth and swimming velocity of the fish. To control the stress of the fish and their feeding behavior, it is necessary to monitor the swimming depth and swimming velocity. Our system can determine the swimming depth and estimate the changes in the swimming velocities. The LDR employed in this system are the NSL 19M51 form Advanced Photonix [47]. The resistance of this LDR varies from 5 kΩ to 20 MΩ. One limitation of our system is that it only can operate when the halogens are turned on.

Nevertheless, as we pretend to monitor the behavior for feeding purposes, the feeding is only provided when the halogens are turned on. During the night, if an abnormal situation occurs and causes stress on the fish resulting in changes in their swimming depth and/or velocity it will not be detected by our system.

**Figure 8:**
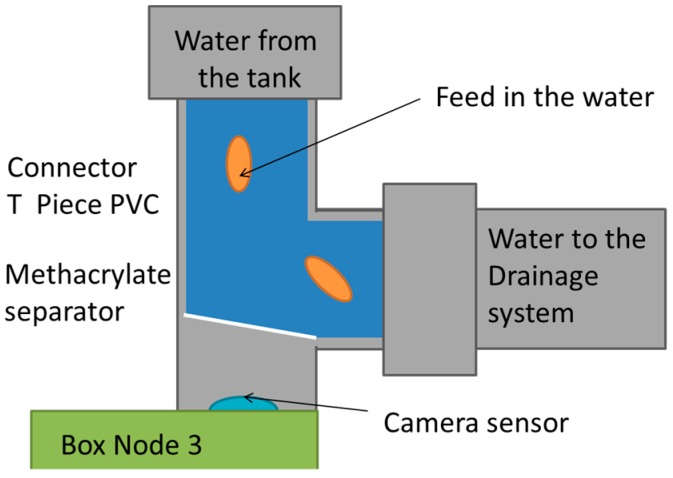
Detail of the location of feed fallen detector.

The sensor to control the falling pellets is detailed in this paragraph. There are different options for pellet detection. While some authors use acoustic methods others use methods based on cameras. In our proposal, a camera will be used to obtain pictures of the pellets in the effluent pipe. Using the same process shown by J. Marin et al. in [48] we will obtain the histograms. Then, we can quantify the amount of pellets according to the number of pixels with a selected value of brightness. The camera will be placed at the bottom of the water drainage system. It will be necessary to change the elbow pipe by a T shaped one and add a Methacrylate separator as shows Figure 8.

#### 3.2.4. Other Sensors

Finally, the humidity level inside the node boxes must be monitored. The humidity node is used to activate an emergency turn off in the BN to prevent further damages caused by the water. It is necessary to consider that the BN2 will be placed inside the water tank and if any damage is caused in the O-Ring of the box the water can enter inside the box. Moreover, in the environment of the production room the level of relative humidity in the air is very high. Thus, the NB1 and 3 also must be monitored. The humidity node is the same sensor employed in [49].

### 3.3. Node

**Figure 9:**
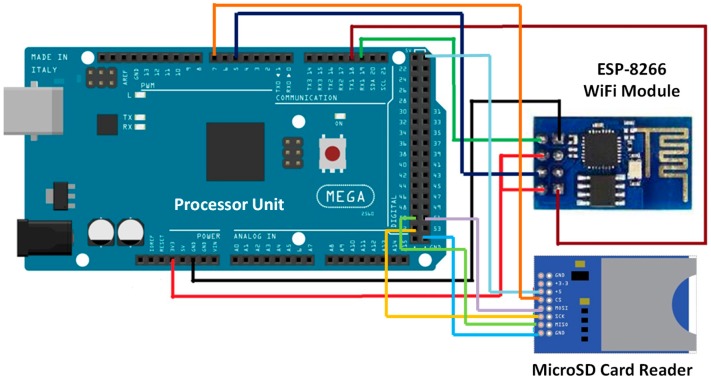
Compatible module with Arduino Mega 2560 connected to WiFi module and MicroSD card reader.

In this subsection, the employed node and its configuration are detailed. To collect the data from all sensors, we have selected a compatible Arduino Mega 2560 module. This microprocessor board has 54 digital input/output pins, 16 analog inputs, 4 UARTs (hardware serial ports), 16 MHz crystal oscillator, USB connection, power jack, In-Circuit Serial Programming (ICSP) connector and reset button. In addition, this compatible Arduino Mega module can be supplied from our PC by means of a USB cable or by an external power supply (9 up to 12VDC). However, one of the main drawbacks of this board is that it does not have any type of wireless interface to integrate it in a WSN and the storage capacity of data from the sensors is limited. To solve this problem, we use a Wi-Fi module ESP8266 ESP-01 [50] and a microSD card reader which will be connected to our microprocessor module as Figure 9 shows.

Another important issue to deal when implementing the module is how to collect the signals from the different LDRs in the node of BN1. In this node the 27 used LDRs must be connected. Moreover, the sensors of humidity, water level, workers presence and illumination are connected too. This issue does not appear in the other nodes. To this end, we select an analog 16 × 1 multiplexer (MUX). In this case, the Multiplexer 74HC4067 [51] will be chosen. Because we need to collect data from 27 LDRs, it is needed to use two 16 × 1 MUX. Finally, to join the signals from both, we will use a new 2 × 1 MUX [52]. Whit this, we will be able to gather data from 30 analog devices.

The resulting operation of this combination of multiplexers works as follows. On the one hand, we have a series of input signals, in our case 32 analog inputs (from AI0 to AI32), which are controlled by the control signals (from C0 to C5). As a function of the control signals, we will select an input signal to drive it to the output. Finally, the STROBE signal enables or disables the multiplexing.

## 4. Results and Discursion

In this section, the results are shown. First, the results of the calibration of water quality sensors are presented. Following the results of sensors for tank monitoring and fish behavior monitoring are presented.

### 4.1. Results of the Water Quality Sensors

In this subsection, the results of the calibration of the employed sensors for water quality monitoring are shown. In some cases, as turbidity or temperature sensors, calibrations have already been carried out that relate the environmental parameter and the resistance of the electronic component. Nevertheless, for operation in the system, it is necessary to obtain the data as the output voltage (*Vout*) that arrives at the node. Moreover, it may be needed to include a voltage divisor to ensure that current in the node is not too high. Thus, it will be necessary to transform the data and to obtain a new calibration equation for the temperature and turbidity sensor. In the case of the conductivity sensor, the same calibration and equations con code that has been used in [38] are employed. Finally, for the oil layer sensor, the calibration shown in [42] is used, the code is presented below.

**Figure 10:**
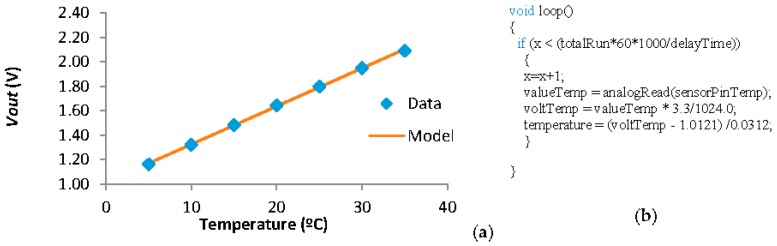
(**a**) Data and (**b**) code of the temperature sensor.

First, for the temperature sensor, based on the data offered by the manufacturer [36] it is possible to extract the expected resistances at different temperatures. A voltage divisor must be used to maximize the difference of *Vout* between the minimum and maximum values of resistance of the NTC. An *R2* of 12 KΩ must be used and the NTC is used as *R1*. We can calculate the *Vout* of the temperature sensor at different temperatures. The data and the mathematical model that follows this data are presented in Figure 10a. The relation between *Vout* and Temperature can be seen in Equation (1). The correlation coefficient of Equation (1) is 0.9995. Considering that the resolution of the node is 10 bits and the maximum Vout that can be registered is 3.3 V, the minimum difference of *Vout* that the node is able to detect is 3.2 mV. Thus, the minimum temperature variation that can be detected by our system is 0.1 °C. The resolution of the temperature sensor is enough for the aquaculture monitoring. In Figure 10b we can see the code used in the node to read the value of the temperature sensor. The formula that relates the temperature and the voltTemp value is extracted from Equation (1).

**Figure 11:**
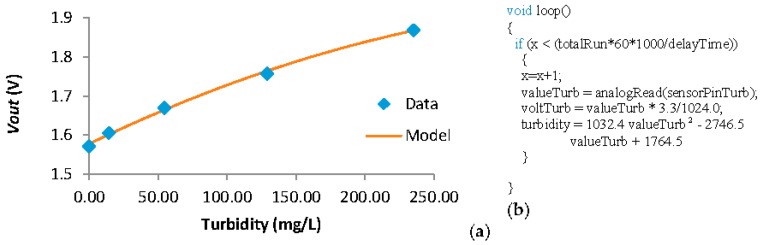
(**a**) Data and (**b**) code of the turbidity sensor.

Following, for the turbidity sensor we use the data shown in [49], where a calibration was already performed. Nonetheless, the data obtained in this calibration must be transformed from the resistance of the IR photodetector to *Vout*. Once more, a voltage divisor must be used. Using the *Vin* of 3.3 V the IR photodetector as *R1* and an *R2* of 6 MΩ we maximize the difference between the maximum and minimum *Vout*. The data after applying the voltage divisor and the mathematical model that adjust to this data are presented in Figure 11a. The correlation coefficient of the mathematical model, Equation (2), is 0.9977. Considering the resolution of the node, the minimum variation of turbidity that can be detected by the turbidity sensor goes from 1.8 NTU in low turbidity conditions to 4 NTU in high turbidity conditions. In Figure 11b, the code introduced in the node to read the *Vout* from the turbidity sensor and transform this voltage into turbidity value is shown. The formula that relates the turbidity and the voltTurb value is obtained from relating the data shown in Figure 11a being the voltage as the independent variable and the turbidity as the dependent variable. The correlation coefficient of this formula is 0.9993.

**Figure 12:**
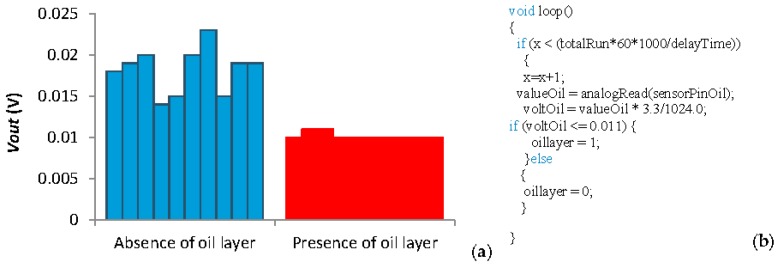
(**a**) Data and (**b**) code of the oil layer sensor.

The calibration of the oil layer sensor is shown now. The data from the oil sensor can be seen in Figure 12a. In this case, we only pretend to differentiate the absence to the presence of oil layer. On the one hand, it is possible to see that when there is no oil layer the average *Vout* is 0.018 V, the minimum value is 0.015 V. On the other hand, when the oil layer is present the average *Vout* is 0.010 V, with a maximum value of 0.011 V. Thus, the threshold value to detect the presence of oil layer is 0.011 V. The code for reading the *Vout* from the oil layer sensor is presented in Figure 12b.

(1) Temperature (°C)=0.0309×Vout (V)+1.1947

(2) $$Turbidity\;\left(\mathrm{NTU}\right)=1764.5+1032.4\times Vout^{2}-2746.5\times Vout$$

### 4.2. Results of the Tank Sensors

**Figure 13:**
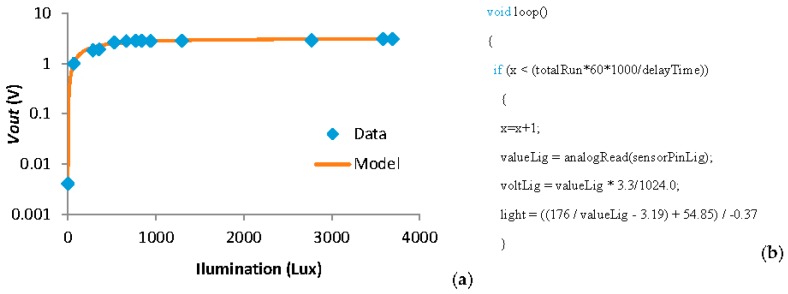
(**a**) Data and (**b**) code of the light sensor.

Following, the results of the calibration from the tank sensors (water level, illumination, and presence sensors) are detailed. The data from the water level sensor is obtained from [38] because the same level sensor is used in this system. The calibration of the illumination sensor is shown in Figure 13a. This calibration was done using a *Vin* of 3.3 V the LDR as *R1* and an *R2* of 2 kΩ. The model presented in Figure 13a presents a correlation coefficient of 0.9855 and can be seen in Equation (3). Considering the resolution of the node, the minimum variation of light that can be detected by the light sensor goes from 0.1 lux in conditions with low illumination to 42 lux when the illumination exceeds the 1500 lux. In the code introduced in the node to read the *Vout* from the light sensor and transform this voltage into illumination in lux value is shown in Figure 13b. The formula that relates the turbidity and the voltLig value is extracted from Equation (3).

(3) $$Light\;\left(lux\right)=3.19+\frac{176}{-54.85-0.37\times Vout\;\left(V\right)}$$

**Figure 14:**
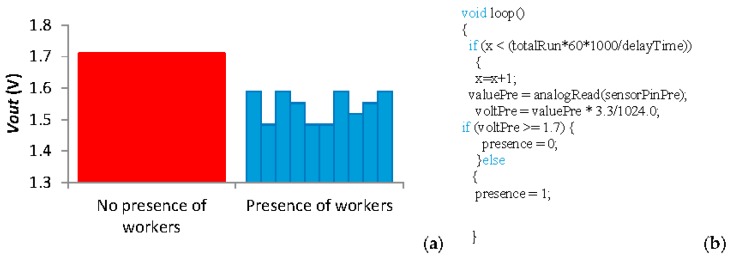
(**a**) Data and (**b**) code of the presence sensor.

The results of the presence sensor are shown in this paragraph. As for the oil sensor, this sensor is not a quantitative sensor, it is a qualitative sensor. It offers information about if there are or there are not people around the tank. Thus, the calibration of this sensor is done comparing the *Vout* of the sensor in two scenarios. In the first scenario, there is no presence of any worker. In the second scenario, there is a worker at 50 cm of the tank. The *Vin* in this test is 3.3 employing an *R2* of 2 MΩ and the IR photodiode as *R1*. The *Vout* in both scenarios is presented in Figure 14a. The values of *Vout* without workers are between 1.5 and 1.6 V, while with the presence of workers the *Vout* increases to 1.7 V. The code of the sensor can be seen in Figure 14b.

### 4.3. Results of the Fish Behaviour Sensors

The results of the fish behavior sensor are shown below. Several tests were done to ensure that this system can correctly detect the fish to determine the swimming depth and estimate the swimming velocity. Furthermore, the camera was tested to detect the presence of feed falling.

**Figure 15:**
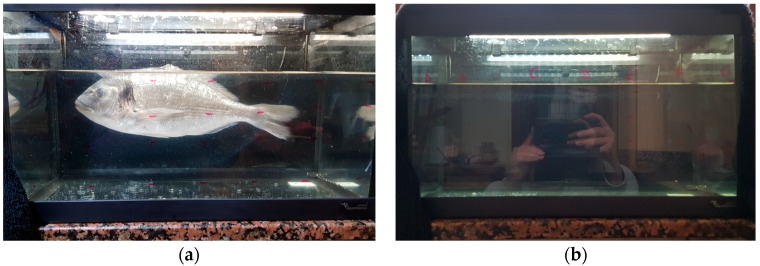
Experiments to demonstrate the operation of fish behavior sensor, preliminary test (**a**) presence and (**b**) absence of the fish.

**Figure 16:**
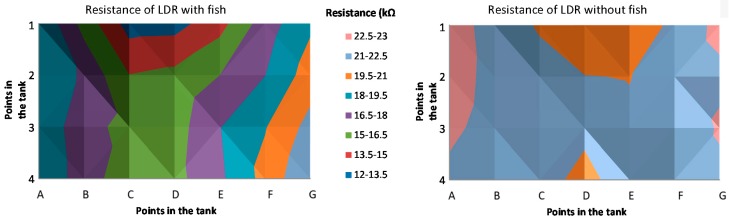
Average LDR resistance in different point with and without the fish.

**Figure 17:**
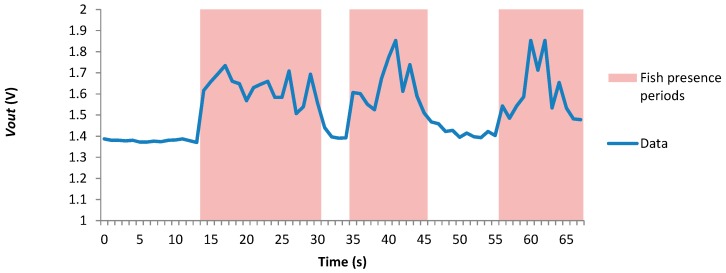
Data gathered by the fish presence sensor.

The first test consists of measuring the resistivity of the employed LDR at different points of an aquarium in the presence (Figure 15a) and absence of a fish (Figure 15b). For this test, a dead adult of *Sparus aurata* L. has been used. It was necessary to use a dead fish is because it is necessary that the fish remains at the same point for the entire experiment. Three repetitions of each measure have been done. In the aquarium, a matrix of 7 per 4 measure points has been set as can be seen in Figure 15a as red dots in the aquarium. The resistance of the LDR has been measured with a multimeter. The average resistance of the LDR in each point of the matrix with and without the fish is presented in Figure 16a,b. It is possible to see that, with the resistance of the LDR we can differentiate when there is or there is not a fish in the aquarium. On the one hand, the lowest resistance when there is a fish is 12.9 kΩ. On the other hand, the lowest resistance value when the fish is present is 19.7 kΩ. Thus, the use of LDR for detecting a fish is demonstrated. Once, it has been demonstrated that with the LDR is possible to determine the presence or absence of fish, we need to gather data with living fish. In the first test with a tank, unique LDR was introduced at 60 cm depth. The *Vout* of the presence sensor is shown in Figure 17. A *Vin* of 3.3 V and an *R2* of 41 KΩ was used. During the first 10 s of measurement, the fish were kept far from the LDR in order to have data without the fish presence. The movement of the fish has been recorded with a camera to compare the data of the sensor with the moments that the fish were close to the detector. The time when the fish were close to the LDR are considered as fish presence periods and are colored in red in Figure 17. The *Vout* of the presence sensor is correlated with the presence of fish. When the fish are present the *Vout* increases to reach values of 1.85 V. The average *Vout* when the fish are present is 1.63 V. In the periods when the fish are not present, the minimum *Vout* is 1.37 V and the average 1.39 V.

**Figure 18:**
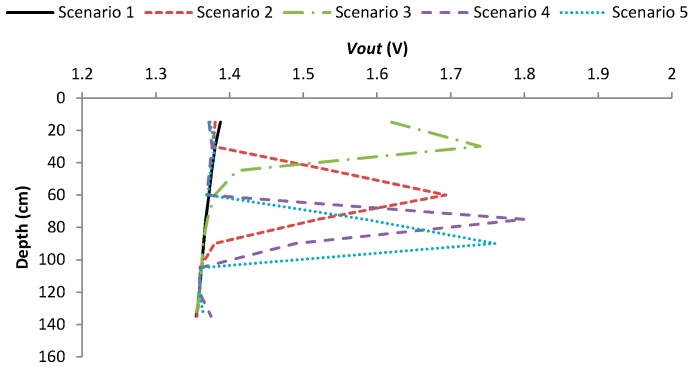
Data gathered by the fish presence sensor.

The next two experiments are performed to test the system for fish detection. First, 9 LDR were introduced in the previous tank with the same fish employed in previous experiment. The *Vout* of each sensor for fish detection is presented in Figure 18. The data of 5 different moments or scenarios are presented. In scenario 1, the fish were not present in the tank. In the rest of scenarios, it is possible to identify the swimming depth by analyzing the *Vout* of the sensor. The maximum value of *Vout* corresponds to the average fish swimming depth. Consequently, it is possible to see that the data of fish detection can be used to determine the fish swimming depth. Unlike with the other sensors where the *Vout* was transformed into the value of the sensed variable before being sent, with this sensor the *Vout* is the sent data. In the database, a smart algorithm will be applied.

**Figure 19:**
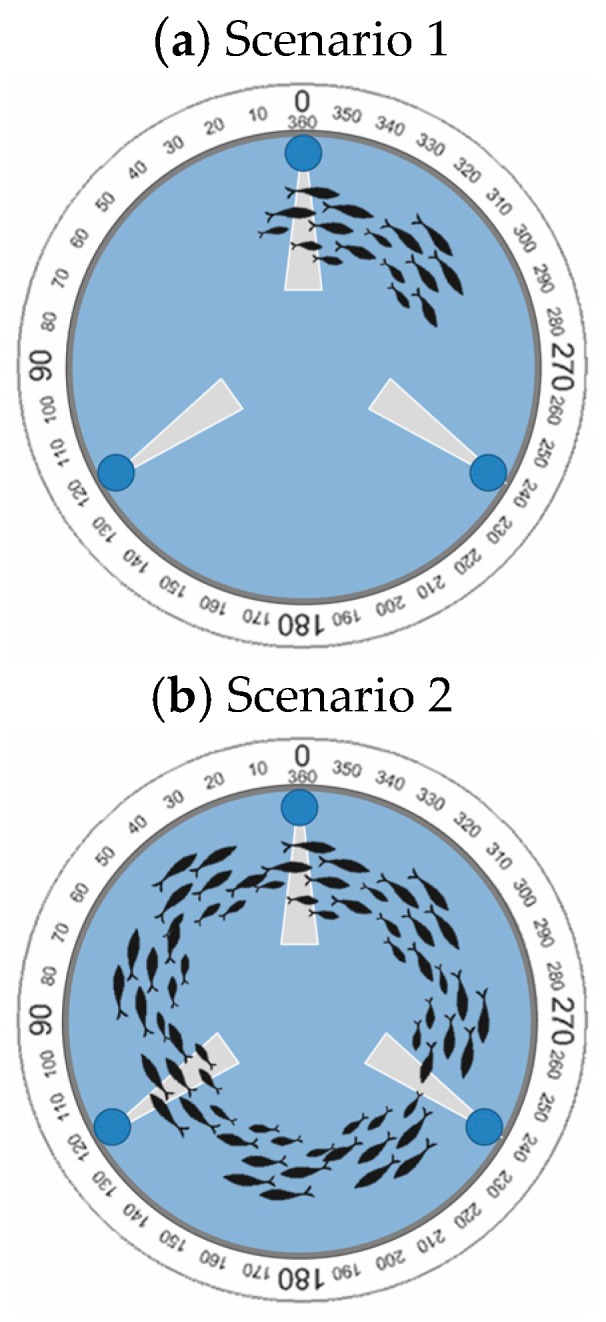
Fish shoal distribution in tested scenarios and LDR location.

**Figure 20:**
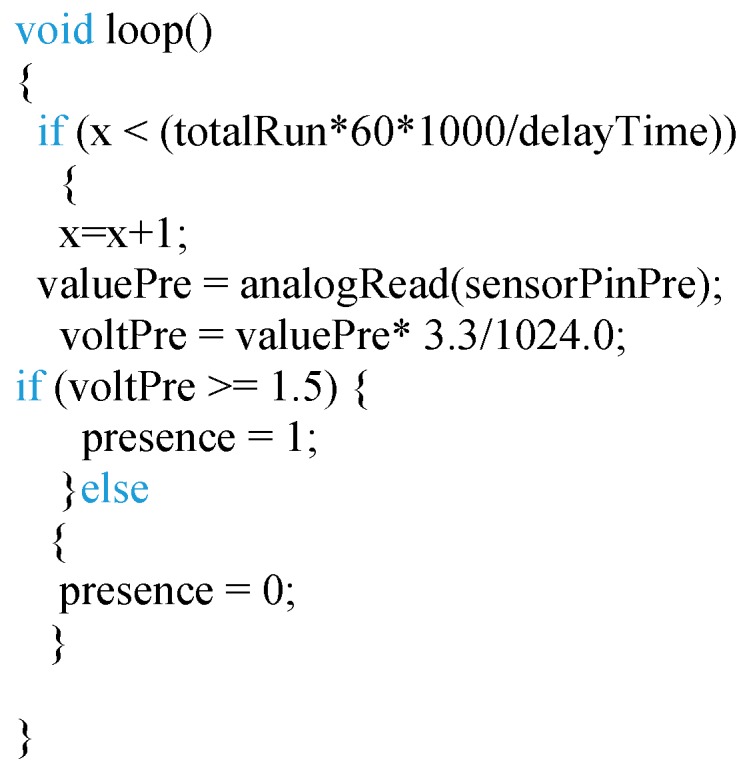
Code for presence sensor.

**Figure 21:**
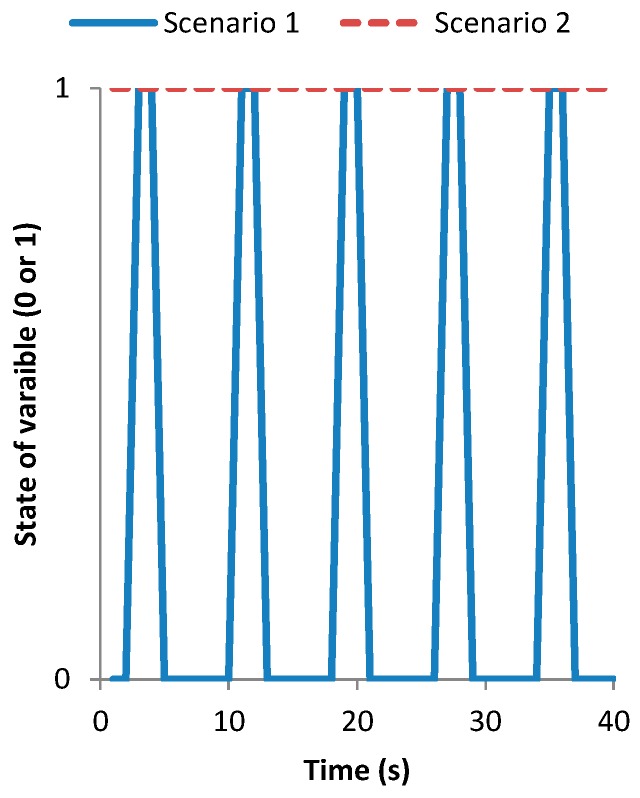
State of variable fish presence of one sensor in different scenarios.

The last experiment was carried out to estimate the fish swimming velocity. Two scenarios were considered for this test. The first one is when there is low fish density and a small shoal is formed. In this case, the shoal does not form a ring, see Figure 19a. The second scenario is when there is a higher density and a bigger shoal is formed. In this case, the shoal forms a ring; see Figure 19b. In this case, instead of representing the *Vout* of the sensor we process the data with the code (see Figure 20) to read this sensor. As this presence sensor gives qualitative data a threshold value must be set. In the database, it is considered that the fish are present if the *Vout* is higher than 1.5 V, and the state of variable is 1. Consequently, when the value of the *Vout* received is lower than 1.5 V the state of variable is set on 0. In the first scenario, Figure 19a, the data after being transformed by the algorithm can be seen in Figure 21. In this case, the state of the variable fish presence changes from 0 to 1 periodically, indicating the pass of the fish shoal close to the presence sensor. Thus, it is possible to estimate the shoal mean velocity (MV) by considering that the shoal swims in a circle and estimating that the diameter of this circle is 10% of the tank diameter. The MV will therefore be given by Equation (4):

(4) $$MV\;\left(\frac{m}{s}\right)=\frac{\left(\emptyset Tank\;\left(m\right)\times 0.9\right)\times \pi }{Time\;between\;intervals\;\left(s\right)}$$

**Figure 22:**
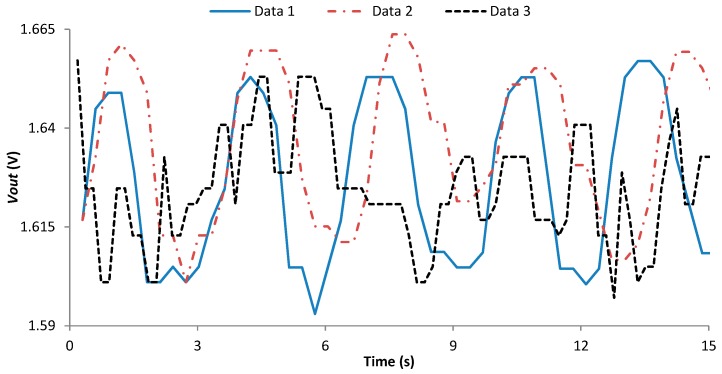
Data gathered by the fish presence sensor.

Nevertheless, in scenario 2 it is not possible to measure the fish MV with this methodology. In this case, it is possible to estimate the variations in the velocity using the variations in the *Vout*. Different fish velocities require different frequencies of propulsion. The greater the movement, the faster the changes in illumination received by the LDR. In this case, it will be impossible to establish the MV; but the system can give information about if the MV increase or decrease. Three tests were done in a tank with fish swimming at different velocities. The data from scenario 1 and scenario 2 corresponds to fish swimming at slower velocity than in scenario 3. The frequency of sensed data in these experiments is the maximum available by the system, 1 data each 0.33 s. The *Vout* data of the three scenarios are presented in Figure 22. We can see that in scenario 1 and 2 there is an oscillation similar to a sine pattern between the maximum and minimum *Vout* values almost constant. However, in scenario 3 this sine pattern is not visible. This is because the flashes produced by the fish scales illuminate the LDR for very short periods. The LDR needs a little time exposed to the light to reach the maximum value and in scenario 3 the flashes are shorter than this period. Then, the LDR is not able to reach the maximum value reached in the other two scenarios. The best way to differentiate between scenarios is to consider the number of peaks in the *Vout* that appear over 15 s. While in scenarios 1 and 2 the number of peaks is equal to 5 in scenario 3 the number of peaks is 11. Therefore, the number of peaks in *Vout* over 15 s is a good indicator of MV (IMV); see Equation (5). When the MV increases the number of peaks will increase.

(5) $$IMV=\frac{n{}^{\circ}\;of\;peaks}{time\;\left(s\right)}$$

**Figure 23:**
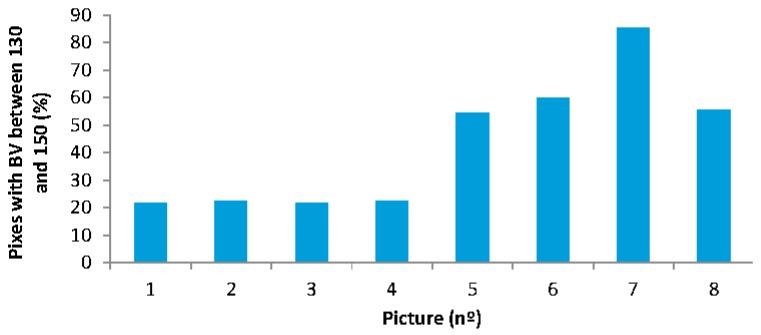
Data gathered by the feed falling sensor.

Finally, the results of the sensor employed to detect the feed falling are presented. For the calibration of this sensor, pictures of the drainage tube without pellets and with pellets are analyzed. The histograms of those pictures were obtained using a Matlab library. First, we analyze the red, green, and blue histograms to compare the values of the pictures without feed and pictures with feed. The blue histogram is the one that shows the highest differences between both situations. Specifically, in the blue histogram, the region with the highest differences, the RI, is the region of blitheness values (BV) between 130 and 150. The ∑RI of different pictures as the % of pixels in the RI can be seen in Figure 23. It is possible to see that in Figure 23, pictures 1 to 4 present values lower than 25% while pictures 5 to 8 have values higher than 50%. Thus, the threshold for this sensor will be the ∑RI value of 30%.

### 4.4. Comparison with Other Systems and Price of the Employed Components

At this point, we are going to perform a comparison between our developed solution and the current systems described in the related work for tanks and ponds, the systems proposed for marine cages are not included. We summarize the characteristics of the current systems and our proposal in Table 1. The main differences are that the other systems monitor between 2 and 4 parameters while our systems are capable to monitor 10 parameters. Moreover, the other proposals employ commercial probes and our system is based on our own sensors. Which makes that the price of the system decreases. In our proposal, we also consider the fish movement among the water quality monitoring. Finally, in our solution we showed the location of the sensors in the tank. The majority of the papers do not pay attention to the location of the sensors.

As a final point, the price of the components to create this WSN for one tank is shown in Table 2. The price of employed sensors is 47.44 € and the price of the nodes and their accessories is 40.22 €. The most expensive item is the workers presence, this sensor has a cost of 15.23 €. All the sensors for water quality have a price of 3.11 €. The system for fish detection cost 16.5 € and the camera for feed detection 5.35 €. To this price some minor costs as boxes for the nodes or plexiglass tubes must be add. The cost of this system can be assumed by the fish farmers, at least in the tanks with the most sensible and/or expensive fish are kept at the beginning and expand the system in the future. The system presents high scalability and only the need components must be bought.

## 5. Conclusions

In this paper, a low-cost WSN for aquaculture monitoring has been shown. The system can control the changes in water parameters, tank state and fish behavior during the feeding process. The monitored water parameters are the temperature, conductivity, turbidity, and the presence of oil layer over the water. The tank state parameters monitored by the system are the illumination, the water level, and the presence of workers. Finally, the fish behavior is monitored with the fish swimming depth sensor and velocity sensor, and the sensor that let us know the amount of feed falling. The topology and architecture have been detailed. Low-cost sensors have been designed, calibrated, and deployed. Smart algorithms were designed to diminish the use of energy in the data transference from the node to the database.

The sensors shown in this paper can be used to improve the efficiency of the aquaculture and the cost of the entire described system is less than 90 €. The sensors are composed of simple electronic components. All the sensors have been calibrated and their suitability for the aquaculture monitoring has been exposed. The greatest efforts have been accomplished with the fish presence sensors and with the feed falling sensor. With the fish presence sensor, several tests were done. First, we performed tests to evaluate the suitability of LDRs to detect the presence of fish were done. Then, different tests were done to determine the presence of fish in different scenarios to calculate the fish depth and fish velocity. With the feed falling sensor, tests in real scenarios were done to gather images of the water drainage system with and without feed. The results show that with the blue band of the picture it is possible to distinguish the presence of feed with ∑RI.

Regarding future work, we will estimate the data network requirements such as the number of APs needed in the production rooms. Moreover, we will study the way to send the alarm messages to the workers directly. In terms of sensors, we expect to create a low-cost dissolved oxygen sensor. In addition, with the implantation of this system, it will be possible to apply data mining techniques and artificial intelligence to predict the feed needed in each tank. We are planning to develop the platform to view the data as it was shown in [53] with LabVIEW and include artificial intelligence for diagnosis with the data from the database as it was done in [54]. We also plan to apply this system to underwater cages as it was done in [22].

## Figures and Tables

**Table 1:** Summary of characteristics of the current system and our proposal.

Paper	[12]	[13]	[14]	[15]	[16]	[17]	[18]	[19]	[20]	[21]	Our Proposal
Communication technology:	ZigBee	ZigBee	ZigBee	N.I.	ZigBee	ZigBee	LR-WPAN	N.I.	ZigBee	N.I.	WiFi
Applied on:	Tanks	Tank	Tanks	Ponds	Ponds	Tanks	Ponds	N.I.	Tank	Ponds	Tanks
Considers the location of the sensor?					x						x
Develop their own sensors?											x
Store information?	x	x	x	x	x	x	x	x	x	x	x
Send alarm?	x	x		x		x	x	x		x	x
pH		x	x	x	x	x	x		x		x
Water level		x			x						x
Temperature	x	x	x	x	x	x	x	x	x	x	x
Pressure	x										
Dissolved oxygen	x	x		x	x	x	x	x	x	x	
Conductivity			x			x		x			x
Ammonia									x		
Others											x
Fish monitoring											x
Total of monitored parameters	3	4	3	3	4	4	3	3	4	2	10

**Table 2:** Cost of the developed system for each tank.

Purpose			Item	Unitary Cost (€)	Units	Cost (€)
Sensors	Water quality	Temperature	NTCLE413E2103F520L	0.62	1	0.62
		Conductivity	-	0.48	1	0.48
		Turbidity	TSHG6200 IR LED	0.75	1	0.75
			BPW83 IR photodetector	0.38	1	0.38
		Oli layer	VLHW4100 White LED	0.36	1	0.36
			BPW41N IR photodetector	0.52	1	0.52
	Tank	Water level	GP2Y0A02YK0F level sensor	15.23	1	15.23
		Workers presence	TSHG6200 IR LED	0.76	1	0.76
			BPW83 IR photodetector	0.38	1	0.38
		Light	NORPS-12 LDR	1.76	1	1.76
	Others	Fish presence	NSL 19M51 LDR	0.55	30	16.5
		Feed falling	Jtron OV7670 300KP VGA	5.35	1	5.35
		Humidity	HCZ-D5-A Humidity sensor	1.45	3	4.35
Node	Node		Node compatible with Arduino	6.7	3	20.1
	Memory system		Micro SD Card reader	2.19	3	6.57
			Micro SD Card 4 Gb	0.82	3	2.46
	Transmission system		ESP8266 WiFi module	1.58	3	4.74
	Multiplexors		CD74HC4067 16-Channel MUX	0.45	2	0.90
			74LVC1G3157 Single-Pole Double-Throw Analog Switch	0.45	1	0.45
	Other		Resistances	0.5	10	5
Total						87.66
